# High Iron and Iron Household Protein Contents in Perineuronal Net-Ensheathed Neurons Ensure Energy Metabolism with Safe Iron Handling

**DOI:** 10.3390/ijms23031634

**Published:** 2022-01-31

**Authors:** Anja Reinert, Tilo Reinert, Thomas Arendt, Markus Morawski

**Affiliations:** 1 Institute of Anatomy, Histology and Embryology, Leipzig University, An den Tierkliniken 43, 04103 Leipzig, Germany; 2 Paul Flechsig Institute of Brain Research, Leipzig University, Liebigstraße 19, 04103 Leipzig, Germany; tilo.reinert@medizin.uni-leipzig.de (T.R.); thomas.arendt@medizin.uni-leipzig.de (T.A.); markus.morawski@medizin.uni-leipzig.de (M.M.); 3 Max Planck Institute for Human Cognitive and Brain Sciences, Stephanstraße 1A, 04103 Leipzig, Germany

**Keywords:** brain, iron, iron proteins, perineuronal net, neurodegeneration, transferrin, transferrin receptor, ferritin H/L, DMT1, MTP1, cellular quantification

## Abstract

A subpopulation of neurons is less vulnerable against iron-induced oxidative stress and neurodegeneration. A key feature of these neurons is a special extracellular matrix composition that forms a perineuronal net (PN). The PN has a high affinity to iron, which suggests an adapted iron sequestration and metabolism of the ensheathed neurons. Highly active, fast-firing neurons—which are often ensheathed by a PN—have a particular high metabolic demand, and therefore may have a higher need in iron. We hypothesize that PN-ensheathed neurons have a higher intracellular iron concentration and increased levels of iron proteins. Thus, analyses of cellular and regional iron and the iron proteins transferrin (Tf), Tf receptor 1 (TfR), ferritin H/L (FtH/FtL), metal transport protein 1 (MTP1 aka ferroportin), and divalent metal transporter 1 (DMT1) were performed on *Wistar* rats in the parietal cortex (PC), subiculum (SUB), red nucleus (RN), and substantia nigra (SNpr/SNpc). Neurons with a PN (PN^+^) have higher iron concentrations than neurons without a PN: PC 0.69 mM vs. 0.51 mM, SUB 0.84 mM vs. 0.69 mM, SN 0.71 mM vs. 0.63 mM (SNpr)/0.45 mM (SNpc). Intracellular Tf, TfR and MTP1 contents of PN^+^ neurons were consistently increased. The iron concentration of the PN itself is not increased. We also determined the percentage of PN^+^ neurons: PC 4%, SUB 5%, SNpr 45%, RN 86%. We conclude that PN^+^ neurons constitute a subpopulation of resilient pacemaker neurons characterized by a bustling iron metabolism and outstanding iron handling capabilities. These properties could contribute to the low vulnerability of PN^+^ neurons against iron-induced oxidative stress and degeneration.

## 1. Introduction

No other organ than the brain constantly needs readily available iron in a regional, cellular and age-dependent manner [1]. Iron is essential for numerous enzymes, for ATP production, myelination and synthesis of DNA, RNA, proteins and neurotransmitters [1,2,3]. A failure to meet this demand for iron can result in persistent neurological and cognitive dysfunction [1]. On the other hand, increased iron levels and iron accumulations in specific brain regions and cells are hallmarks for more than 15 neurodegenerative diseases including Alzheimer’s and Parkinson’s disease [1,2,3,4,5,6,7,8,9]. In healthy brains, iron also accumulates as a normal process of aging. This is caused by a slow brain iron turnover and an influx being higher than the efflux. This process was investigated in a longterm-study on healthy rats [7].

Oxidative stress is a pathological key feature of neurodegeneration and aging processes. Excessive free iron increases the risk to generate highly reactive radicals such as hydroxyl radicals via the Fenton reaction. This stimulates oxidative stress and causes damage to DNA, proteins, and lipids, eventually leading to cell death. Iron-related cell death is specified as ferroptosis, a recently discovered mechanism of iron-mediated cell death distinct from apoptosis [10,11]. Under healthy conditions detrimental effects of the Fenton reaction are mitigated by antioxidants (e.g., glutathione, superoxide dismutase), which, however, are mainly localized in astrocytes and microglial cells.

Interestingly, there is a subpopulation of neurons that has been shown to be less vulnerable against iron-induced oxidative stress and degeneration: neurons ensheathed by a perineuronal net (PN) [12,13,14,15,16,17]. The PN is a specialized form of extracellular matrix that covers the soma, dendrites and axon initial segment of the neuron and receives its mesh-like structure due to permeating presynaptic boutons [18,19,20]. Despite the PN’s effects on the extracellular space due to its physicochemical properties [21], a PN also reduces the probability of lipofuscin accumulations in PN-ensheathed (PN^+^) cortical neurons compared to neurons without a perineuronal net (PN^−^) [13]. The effect was particularly pronounced in PN^+^ interneurons that typically possess a strong PN, while for delicately ensheathed pyramidal cells, the protective effect was considerably lower. PNs seem to prevent accumulations of lipofuscin by reducing the oxidative stress.

PNs were also resistant to microglia-induced oxidative burst in rat hippocampal neurons [22]. Furthermore, cortical neurons surrounded by a PN are virtually not affected by neurofibrillary tangles in AD [23,24]. Similar observations for PNs and pathological hallmarks in AD, neurofibrillary tangles and plaques, were also shown in other studies, including aged bisons, cortical rat cell cultures and the AD mice model Tg2576 [24,25,26,27]. The occurrence of PNs seems to account for the less affected motoric and primary sensory brain regions in AD. In these regions, PNs are especially numerous [23]. The regional correlation also appears in the opposite effect, the entorhinal cortex having only a small number of PN-ensheathed neurons is early and heavily affected in AD [28]. In PD the degenerating dopaminergic neurons in the substantia nigra pars compacta are never surrounded by a PN [29].

PNs consist of the chondroitin sulfate proteoglycans aggrecan, versican, brevican, neurocan and phosphacan, hyaluronic acid, tenascin-R, and link proteins [30,31,32,33,34,35]. The PN’s molecular structure allows for variability and durability, which is indispensable for neuronal and synaptic plasticity and memory [20,21,36,37,38]. In cortical areas, PNs are often around highly active, fast-firing neurons (parvalbumin and Kv3.1b positive), thus it is believed to be a rapid, local buffer for excessive cations regulating the local ion homeostasis [17,25,39,40,41,42,43].

In addition to this physiological mechanism, the PN is a highly effective iron scavenger. Due to its fixed high negative charge density, it can bind free iron up to 200 times compared to other brain structures [21]. Thus, microinjected iron chloride into the brain cortex of mice resulted in iron-induced oxidative stress and ferroptotic PN^−^ neurons, but PN^+^ neurons were more likely to survive [15,16]. This neuroprotective effect was shown to be directly mediated by the PN, as in PN-component knockout mice the effect was reduced. The PN is therefore an effective protection against iron-induced oxidative stress as it binds free or loosely bound iron [44,45,46]. It might act as an internal iron chelator. Shooting for the same, externally introduced chemical iron chelators are tested as therapeutics for iron-associated neurodegenerative diseases in several clinical trials [47,48,49]. The results are reasonably good, but the breakthrough is yet missing. A reason could be the still missing profound understanding of the mechanisms that regulate metal homeostatic processes [50,51].

The brain iron homeostasis relies on a precise regulation and interplay of multiple iron proteins. The neuronal iron import is mediated by the transferrin–transferrin receptor complex (Tf-TfR) and by the divalent metal transporter 1 (DMT1). Tf is an effective protector against iron-mediated cell damage due to its negative reduction potential [52]. One Tf molecule can bind two Fe^3+^ ions with high affinity. The TfR is a transmembrane protein that binds two iron-loaded Tf molecules. The Tf-TfR complex is initialized by endocytosis, and iron is released from the complex by a ph drop in the endosome. The membrane-bound protein DMT1 mediates the iron transport from the endosome into the cell lumen. Tf and TfR are recycled by exocytosis. DMT1 also enables the iron import from the outer to the inner site of the cell independently from the Tf-TfR import. Since DMT1 only transports divalent metal ions [53,54], the iron needs first to be reduced. This is performed by a ferrireductase as duodenal cytochrome B (DcytB). Even though the name implies DcytB to be located in the duodenum, the mRNA of DcytB was also proven to be present in rodent brain [55]. The metal transport protein 1 (MTP1) is the only known intracellular iron exporter in mammals and is expressed in several neuronal types [56,57,58]. Ferroxidases (e.g., ceruloplasmin, hephaestin), mRNA-binding iron regulatory proteins (IRPs) and the hormone hepcidin are additionally involved in brain iron homeostasis, but were not investigated in this study.

The intracellular storage of iron is mainly done by ferritin (Ft). One ferritin molecule has the capacity to store 5000 iron atoms in a mineral core which, however, is filled in average only half as a porous sphere [59]. Under physiological conditions in the human cerebral cortex and the cerebellum, ferritin was found to bind approximately 1500 and 1850 iron atoms, respectively [60]. For rat neurons, we have estimated from measured iron concentrations and published average iron loadings an intrasomal density of 133 ± 25 Ft molecules per μm^3^ [61]. The ferritin molecule is composed of 24 subunits of FtH (heavy chain) and FtL (light chain) in a cell type specific ratio [1,62]. FtH acts as a ferroxidase and is therefore able to oxidize ferrous iron (Fe^2+^) for a rapid uptake of iron ions. FtL instead participates in the assembling of the ferritin core and mainly functions as a long-term storage [62]. The reaction of iron with H_2_O_2_ is effectively prevented by its storage as ferric iron (Fe^3+^) within the ferritin molecule [63].

Interestingly, even though the iron binding ability of the PN and its neuroprotective effect against iron-induced oxidative stress was shown [15,21], the physiological iron concentration of the PN itself is not increased compared to the cytoplasm of the ensheathed neuron [14]. When the highly negatively charged PN has no higher iron concentration than the neuron itself, what happens to the scavenged iron? An indication gives a preliminary study where PN^+^ neurons had more intracellular iron compared to PN^−^ neurons [14].

We hypothesize that PN-ensheathed neurons have a specialized iron household with potent expression of intracellular iron transport and storage proteins capable of safely handling surges of PN-scavenged iron as well as a higher demand due to faster energy metabolism. Iron that is bound to intracellular iron proteins—to be stored, for need or on hold—is no longer available for the Fenton reaction, which reduces the local iron-induced oxidative stress of this neuronal subtype.

The objectives of this study are to quantify the intraneuronal iron concentrations as well as the amount of iron transport and iron storage proteins in PN^+^ and PN^−^ neurons in different brain regions with the aim to find the distinct mechanism that explains the lower vulnerability of PN^+^ neurons. The applied methods for cellular analysis are scanning particle-induced X-ray emission (µPIXE) for quantitative element mapping and immunohistochemistry (IHC) quantified by slide-based cytometry (SBC). Supporting regional data on iron protein and mRNA content acquired in PN-rich regions was done with quantitative Real-Time-PCR (qRT-PCR) and Western blotting (WB). The investigated iron proteins are transferrin (Tf), transferrin receptor 1 (TfR), ferritin H (FtH), ferritin L (FtL), divalent metal transporter 1 (DMT1; synonym: divalent cation transporter 1, DCT1), metal transport protein 1 (MTP1; synonyms: ferroportin 1, IREG1) and duodenal cytochrome B (DcytB). The investigated brain regions are parietal cortex (PC), subiculum (SUB), red nucleus (RN), substantia nigra pars compacta (SNpc) and reticulata (SNpr), and entorhinal cortex (EC), the latter one as a PN-poor reference region.

This combined approach of studying iron proteins as well as iron concentrations on the cellular level can easily be extended to animal models of iron-associated brain diseases and human brain samples with normal aging or pathological iron accumulations. Those studies will link the cellular iron homeostatic mechanisms to potential biomarkers of iron load in magnetic resonance imaging and cerebrospinal fluid. Furthermore, it may help to provide the necessary information for more targeted therapies.

## 2. Results

### 2.1. Higher Iron Concentrations in PN^+^ Neurons

**Figure 1:**
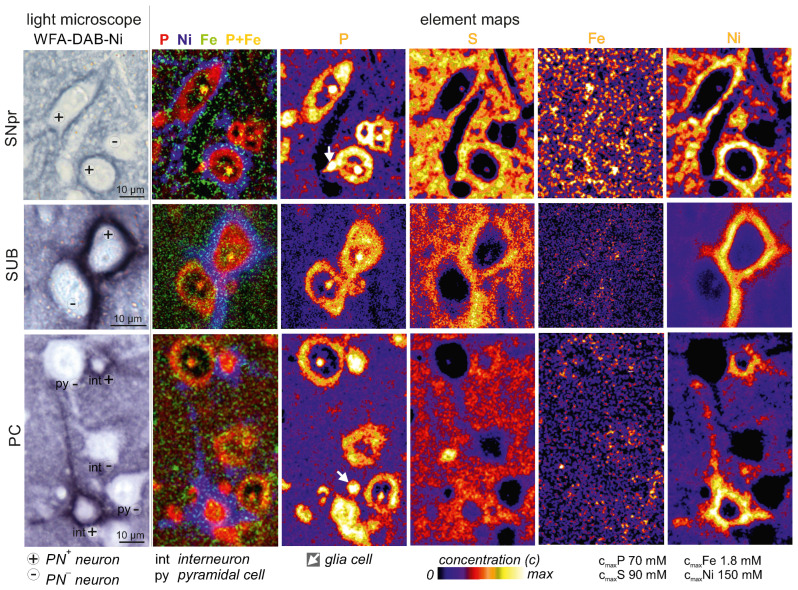
Quantitative element maps of groups of PN^+^ and PN^−^ neurons in the rat brain regions parietal cortex (PC), subiculum (SUB), and substantia nigra pars reticulata (SNpr). PNs appear in the nickel map due to Ni-DAB-enhanced WFA-staining. Intracellular iron concentrations were extracted from manually drawn ROIs.

The quantitative element maps, that were obtained by µPIXE analysis (Figure 1), allow to identify the ROIs, PN^+^ and PN^−^ neurons as well as the PN, and to extract the average iron concentrations therein. High phosphorus content indicates cell somata of neurons and glia cells. Cells were morphologically identified in the P-map. The chondroitin sulfate component of PNs make strong PNs slightly visible in the sulfur map. PNs appear unambiguously in the nickel map due to nickel enhanced DAB-WFA-labelling.

**Figure 2:**
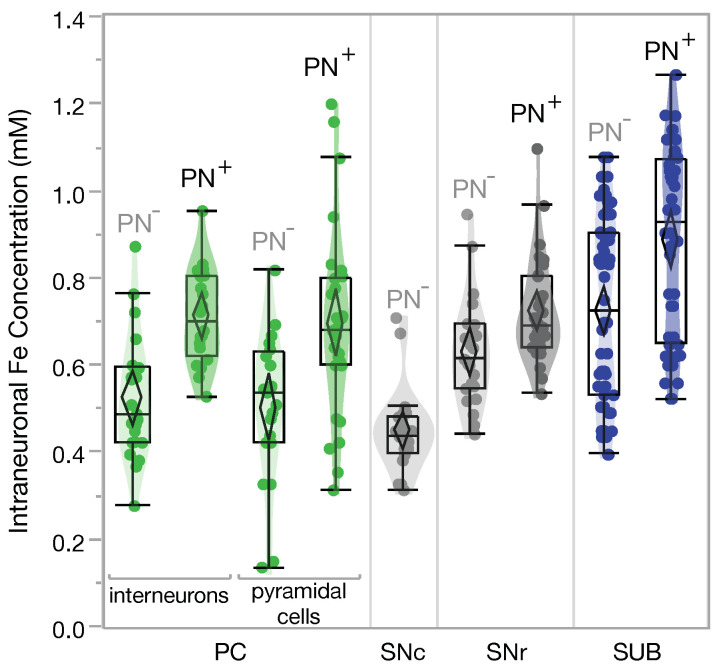
Intracellular iron concentrations of neurons with (PN^+^) and without (PN^−^) a PN in the parietal cortex (PC), subiculum (SUB), and substantia nigra pars reticulata (SNr)/compacta (SNc). PN^+^ vs. PN^−^ *p*<0.01, SNc *p*<0.02 (Student’s *t*-test).

The average intracellular iron concentrations of neurons with and without a PN for the regions PC, SUB, and SN are presented in Figure 2.

For the analysis of PC neurons we further distinguished between interneurons and pyramidal cells since PN^+^ pyramidal cells possess a morphologically different PN [64]. PN-ensheathed pyramidal cells have a 35% higher iron concentration than pyramidal cells without a PN, (0.69 ± 0.05) mM vs. (0.51 ± 0.04) mM. The same results, within the margin of error, were found for PN^+^ and PN^−^ interneurons, 36% more iron in PN^+^ than in PN^−^ interneurons, (0.71 ± 0.03) mM vs. (0.52 ± 0.04) mM. PN^+^ neurons in the SUB have a 23% higher iron concentration than PN^−^ neurons, (0.84 ± 0.03) mM vs. (0.69 ± 0.02) mM. In the SN, the analysis was done separately for neurons in the pars reticulata (pr) and pars compacta (pc). The PN^+^ neurons of the SNpr have a 14% higher iron concentration than the PN^−^ neurons, (0.71 ± 0.03) mM vs. (0.63 ± 0.05) mM. In the SNpc there are virtually no PN^+^ neurons, i.e., all SNpc neurons are PN^−^ neurons. SNpc neurons have with (0.45 ± 0.02) mM the lowest iron concentration among the analysed neurons.

We also analysed the iron concentrations at 77 PNs, 33 within PC, 15 within SN, and 29 within SUB. There was an average 4 ± 2% lower concentration at the PN compared to the soma of the associated neuron (Mean ± SEM, *p*<0.01, paired student’s *t*-test).

**Figure 3:**
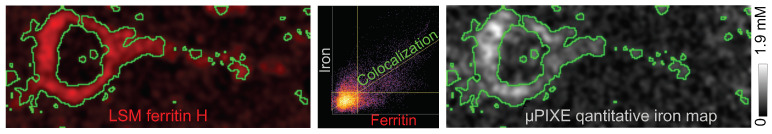
Correlative fluorescence and element microscopy. Co-localization of ferritin H and iron in the cytoplasm of an interneuron from the parietal cortex. Left panel: Cy3 signal from immunohistochemically stained ferritin H. Right panel: µPIXE quantitative element map of iron. Middle panel: Pixel scatter plot of signal correlation between ferritin H and iron. The pixels in the quadrant with the linear correlation define the area of co-localization (regions encircled in green).

**Figure 4:**
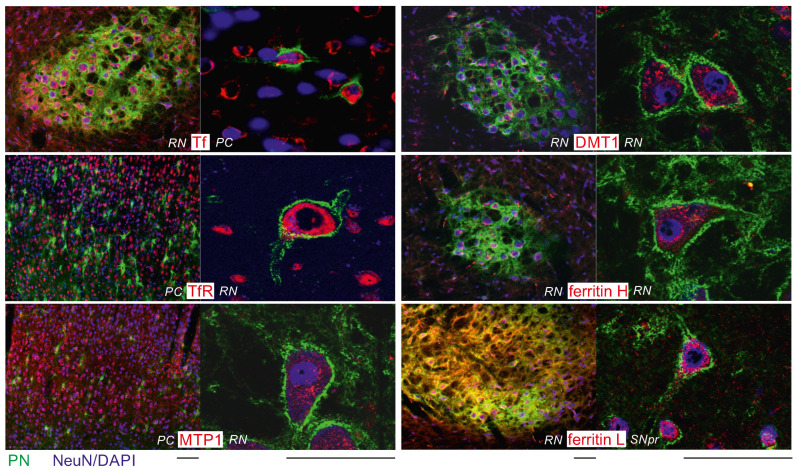
Immunohistochemistry. Brain slices were immunohistochemically stained for iron proteins (antibody, Cy3-labeled) and perineuronal nets (WFA, Cy2). Neurons (NeuN, Cy5), or in case of TfR nuclei of cells (DAPI), were counterstained. The representative images from parietal cortex (PC), red nucleus (RN) and substantia nigra pars reticulata (SNpr) show that all iron proteins can be visualized and are distinctly visible in the cytoplasm of the cells. Ferritin L, which is supposed to be more prominent in glial cells, is also well represented in neurons. A difference in iron protein content of PN^+^ and PN^−^ neurons cannot be distinguished by naked eye, but was revealed by quantification of the immunofluorescence signal intensity using SBC. Scale bar: 50 μm.

Figure 3 shows the co-localization of ferritin and iron in an interneuron from the PC region obtained by correlated laser scanning microscopy and µPIXE quantitative element mapping. Both ferritin and iron are mainly co-localized and most abundant in the cytoplasm of the perikaryon (green encircled region in the LSM and µPIXE maps in Figure 3, Pearson’s correlation coefficient above threshold: 0.51 (Fiji coloc2 plugin)). The karyoplasm is typically low in iron, as it is for iron proteins in general (Figure 4). The nucleolus, however, is known to be an iron hotspot [61].

### 2.2. Intraneuronal Iron Protein Localization

The iron proteins Tf, TfR, FtH, FtL, MTP1, and DMT1 were visualized in immunohistochemically triple-stained brain slices revealing their distribution within the cells (Figure 4). Initially, DcytB was also investigated, but was dropped from further investigations because it showed a fluorescence signal too weak to be analysed. Also the WB signal demonstrated a very weak signature.

All investigated iron proteins are dominantly localized in the cytoplasm of the neurons (Figure 4). Tf, TfR, FtH, FtL, MTP1, and DMT1 were detectable in all NeuN-positive neurons. There was no obvious neuronal prevalence for ferritin H or L. The PN itself does not seem to attract specifically any transport or membrane bound iron protein.

### 2.3. Higher Iron Protein Levels in PN^+^ Neurons

**Figure 5:**
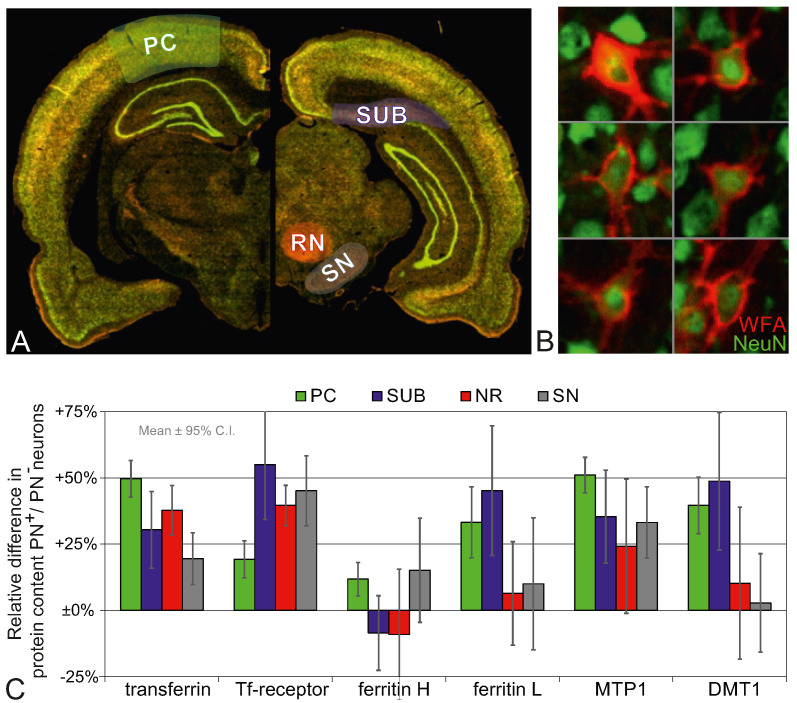
Slide-based cytometry. (**A**) Overview images of rat brain sections with triple-labelling for neurons (NeuN, Cy2 in green), PNs (WFA, Cy3 in orange), and selected iron proteins (Cy5, not shown). The ROIs selected for analysis are highlighted. (**B**) Sample of high resolution images of neurons from PC. The intense Cy3-WFA signal surrounding the NeuN-area (centred) identifies the neuron as PN^+^. (**C**) Ratio of averaged intracellular iron protein contents PN^+^ to PN^−^ from fluorescence intensity data. The dashed line marks the reference value 100% set by the values from PN^−^ neurons. Data bars: Mean ± 95% confidence interval.

Using slide-based cytometry (SBC) the contents of iron proteins in the soma of neurons were quantified from the fluorescence intensity of immunohistochemically bound antibodies. Due to triple-staining, PN^+^ and PN^−^ neurons could be distinguished and analysed automatically in the ROIs (Figure 5A,B). We calculated the ratios of relative iron protein content PN^+^ vs. PN^−^ neurons and the corresponding 99% confidence interval. The results are shown in diagram Figure 5C.

The protein content of Tf, TfR and MTP1 is higher in PN^+^ neurons than in PN^−^ neurons. This result was found in all analysed brain regions. For the other iron proteins ferritin and DMT1 it is less consistent. FtH is slightly higher (12%) in the PC only. In the SN, the result, even though the FtH level is 15% above the PN^−^ level, has a too brought confidence interval to state any robust difference. On the other hand, FtL and DMT1 show a considerably higher content in PC and SUB, but not in NR and SN.

**Figure 6:**
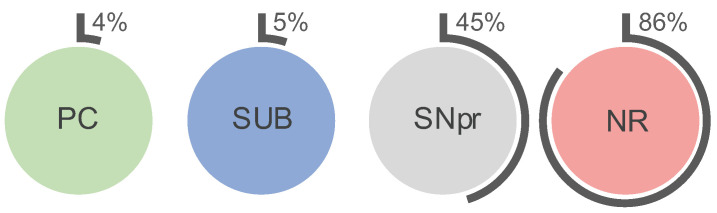
Percentage of PN^+^ neurons as obtained by single cell counting during slide-based cytometry. PN^+^ and PN^−^ neurons were counted in the left and right hemispheres and averaged over 14 brain slices from two rats (seven slices each). Total cell counts within the selected ROIs were 42,593 (PC), 12,301 (SUB), 2958 (NR), and 2726 (SN).

Since SBC identifies individual PN^+^ and PN^−^ neurons, the data also include the cell count within the selected ROIs. In 14 brain sections of two rats, 60,578 neurons were analysed. This allowed us to calculate the percentage of PN^+^ neurons within the analysed regions PC, SUB, NR, and SN. The results are presented in Figure 6.

### 2.4. Higher Iron Protein and mRNA Expression in PN-Rich Brain Tissue

**Figure 7:**
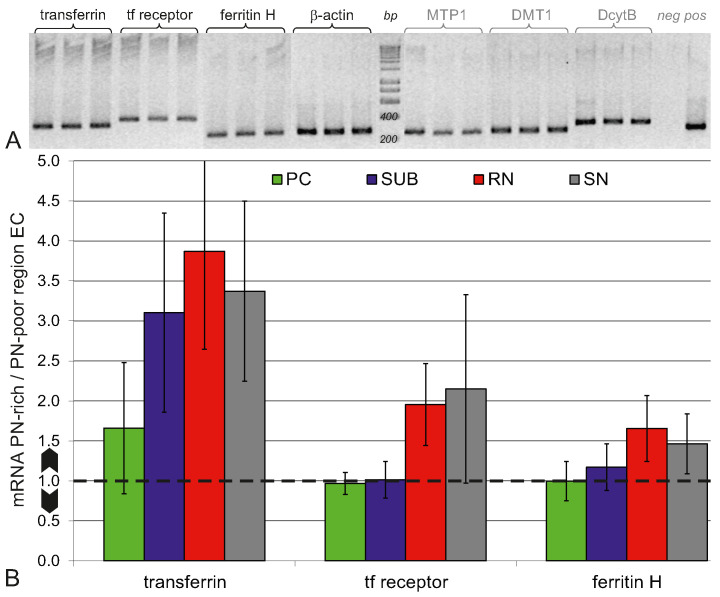
qRT-PCR. (**A**) All cDNA amplification products were verified according to their sequence lengths (data shown for SN). (**B**) Relative quantities of mRNA from PN-rich brain regions with respect to mRNA content in EC (PN-poor region). The dotted line marks the base level ratio of one, i.e., no difference in mRNA expression. Data given as mean ± 95% confidence interval.

We analysed iron protein mRNA expression by qRT-PCR in PN-rich brain regions (SN, PC, RN, SUB) and, for a base value, also in the PN-poor brain region EC. All qRT-PCR products encoding for Tf, TfR, FtH, DMT1, MTP1, DcytB, and β-actin could be verified according to their sequence lengths (Figure 7A). The cDNA amplifications of β-actin, Tf, TfR and FtH achieved their exponential phases during the PCR run within less than 35 cycles and completed with a plateau phase. For DMT1, MTP1, and DcytB the plateau phases were not achieved before the stop cycle 45, which makes their quantification untrustworthy. Therefore, we restricted the quantitative analysis to the iron proteins Tf, TfR and FtH (Figure 7B).

Tf mRNA expression in the PN-rich brain regions PC, SUB, NR, and SN was considerably higher (≥3-fold) than in the PN-poor region EC, most prominent for SUB, NR, and SN. The mRNA expression of TfR was about twice as large in NR and SN then in EC. FtH in NR and SN was expressed about 50% more than in EC. PC and SUB did not show any difference in TfR or FtH mRNA expression.

**Figure 8:**
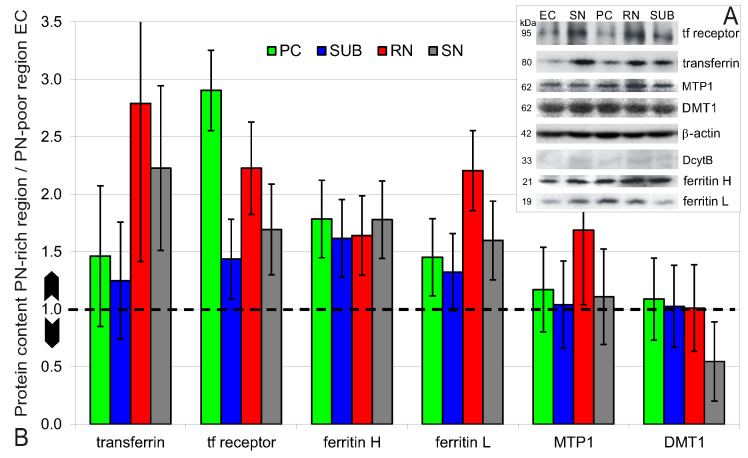
Western blot. (**A**) Iron protein specific antibodies were verified by the molecular weights (kDa) of the target proteins. The DcytB specific antibody showed an insufficient signal which impeded WB analysis. (**B**) Relative quantities of proteins from PN-rich brain regions (PC, SUB, RN, SN) with respect to protein content in the PN-poor region (EC). The dotted line marks the base level ratio of one, i.e., no difference in protein expression. Data given as mean ± 95% confidence interval.

For the WB analysis, antibodies against Tf, TfR1, FtH, FtL, MTP1, DMT1, and DcytB were used. The specificities of the antibodies were proven by the molecular weight of the target proteins (Figure 8A). All iron proteins could be determined except DcytB, which could not be proven satisfactorily with the available antibody. It therefore could not be analysed by WB.

WB revealed a higher expression of TfR, FtH and FtL in the PN-rich brain regions PC, SUB, RN, and SN, while Tf was only higher in RN and SN (Figure 8B). MTP1 and DMT1 are less prominent. The only region with an increased MTP1 expression is the RN. For DMT1 no increase was observed, but a decrease in SN.

For a better overview of the results that demonstrate higher iron protein levels in PN^+^ neurons and for the results from regional protein and mRNA analysis we give a graphical representation of the relation in Table 1.

### 2.5. PN^+^ Neurons/Regions Show High Activity of Cytochrome C Oxidase

**Figure 9:**
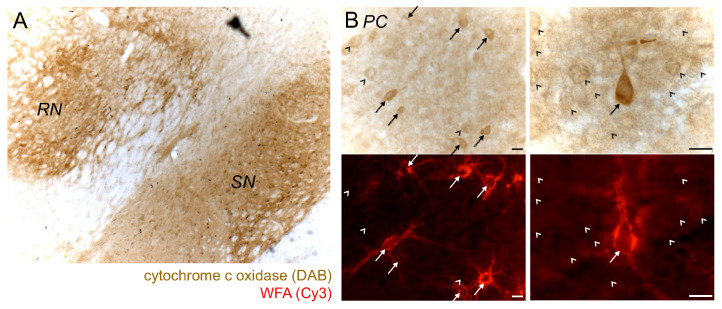
Cytochrome c oxidase enzyme histochemical staining. (**A**) The PN-rich regions red nucleus (RN) and substantia nigra (SN) show increased reaction (DAB-enhanced) for cytochrome c oxidase (CytOx), which is a marker for a high metabolic rate. (**B**) CytOx activity in the parietal cortex (PC). The strongest CytOx reaction is found in PN^+^ neurons (arrows). PN^−^ neurons (arrow head) have a weaker CytOx reaction. Scale bar: 10 μm.

An increased activity of cytochrome c oxidase (CytOx), which is a sign for a high metabolic rate, is pronounced in PN-rich regions (Figure 9A). A cellular analysis was performed on the PC. All PN^+^ neurons stand out with a higher CytOx activity compared to PN^−^ neurons (Figure 9B). There was no PN^+^ neuron with a weak CytOx activity. Although about 10% of the PN^−^ neurons also showed increased CytOx activity, it was generally lower compared to PN^+^ neurons.

## 3. Discussion

### 3.1. Neuronal and Regional Iron Concentrations

The higher iron concentrations in PN^+^ compared to PN^−^ neurons were consistently found in all analysed brain regions PC, SUB, SN. There is no indication that the higher iron content of PN^+^ neurons is related to—despite the regional variations of neuron characteristics—cell types, in particular to interneurons or pyramidal cells, and therefore to the main neurotransmitters GABA and glutamate. Further, the PN^+^ iron content is also not related to the morphological subtype of the PN (according to Wegner et al. [64]), whether it may be thick and sharply contoured, thin, or diffuse.

For the SN, the heterogeneous iron distribution and age-related accumulations are well documented, e.g., in the review of Snyder and Connor [65]. A higher iron content of the SN pars reticulata with respect to the pars compacta has been shown, among others, for healthy human and rat brains [66,67]. However, most of the studies with iron detection or analyses are on regional but not on cellular levels. Though, some have used iron-sensitive histochemical methods to investigate the cellular contributions to the total SN’s iron content. Quantitative in situ studies on the cellular iron content are sparse. Studies from our groups confirm that the highest cellular iron concentrations can be found in glial cells, especially in oligodendroglia, and in neuromelanin containing neurons [61,68,69]. For the young adult rats, that neither have neuromelanin in neurons nor age-related iron accumulations, the neuron specific analysis revealed a plausible intrinsic difference of the intracellular iron content between the neurons in the SNpr and SNpc. The SN neurons with the higher iron content are fast GABAergic neurons of the pars reticulata (30 Hz firing rate) [70]. The slowly firing dopaminergic neurons of the pars compacta (2 Hz firing rate) [71] have a distinctly lower iron content, in fact the lowest iron concentration among the analysed brain regions. Thus, in young age and without neuromelanin, the dopaminergic neurons of the SNpc can be considered iron poor. Further, dopaminergic neurons in the SNpc have thin or sparsely myelinated axons [72]. It may be assumed that a reduction of the energy required for transmission of the action potentials, a profit of myelination [73], is not needed for the low firing rate. It is also worth mentioning that dopaminergic neurons virtually never express a PN, which leaves those neurons bar of the PN’s intrinsic effect with the known increased risk of neurodegeneration in case of higher loads of intracellular iron due to aging or pathological conditions. Therefore, the strategy is to extend the cellular analysis to animal and human brains with iron-associated neurodegeneration in combination with quantitative magnetic resonance imaging [68,69].

Within the cortex, pyramidal cells and interneurons have the same iron concentrations and show the same elevated iron content when ensheathed by a PN. The expression of a PN is not restricted to a particular neurotransmitter type, as cortical GABAergic interneurons and glutamatergic pyramidal cells can both possess a PN. Further, interneurons are surrounded by a distinct, sharply contoured PN and pyramidal cells by a slender PN [64]. Since in a previous study we could show that the PN is a highly effective iron scavenger [21], it is reasonable to assume that a neuron with a thick PN may have a higher iron content than a cell with a thin PN. However, this assumption is not corroborated by the results of this study. The morphological appearance and structure of the PN, i.e., its volume does not correlate with the extend of elevated iron content with respect to the PN^−^ neuron population.

Iron and iron proteins are mainly localized in the cytoplasm. It is obvious that iron and ferritin expectedly show a clear colocalization (Figure 3) because ferritin stores up to 90% of the cellular iron [74,75]. We could show that indeed most of the iron (three quarters) can be traced down to ferritin, which is contained in the microsome fraction of a density gradient centrifugation [61].

While for glial cells, their different iron concentrations can be attributed to oligodendrocytes, astroglia, and microglia [61], for PN^+^ neurons, the only obvious distinction that comes with the different iron content is the occurrence of a PN. Since the PNs have only slightly lower iron concentrations compared to their associated neurons, we assume a close functional relationship between the neuron, iron and its PN. The additional iron within PN^+^ neurons is very likely connected with a higher performance or quantity of iron related proteins that fuel a distinctly faster energy metabolism of the subtype of PN^+^ neurons.

It seems reasonable that the PN is a part of a distinctly different iron household of PN^+^ neurons. This reasoning is corroborated by the results of our iron protein analyses and by analysing the activity of cytochrome c oxidase.

#### 3.1.1. PN and Iron Proteins Protect the Neuron by Handling Excessive Iron

The analyses of iron proteins and mRNA on cellular and regional level revealed for a majority of the 60 data sets an increase of the protein expression in PN^+^ neurons and PN-rich brain regions (Figure 1). On the cellular level, it is conclusive that PN^+^ neurons have a higher iron protein content and expression, as it correlates well with the higher iron content and lower vulnerability of these cells [15]. On first thought, one would expect higher ferritin levels in PN^+^ neurons assuming a need for adequate capacity to safely process excess iron. However, ferritin does not show the ubiquitous increase as Tf, TfR, and MTP1. Since the physiological iron load of ferritin seems to be around half of its full capacity of 5000 iron atoms, there should be enough dynamic storage capacity to effectively buffer occasional surges of excess free iron. Especially when a potent guard of the iron transport and export proteins Tf, TfR, and MTP1 is able to quickly equalize the iron balance.

DMT1 keeps a low profile which is an indication that it may play only a minor role in the faster iron metabolism of PN^+^ neurons. A study from Ke and colleagues suggests that DMT1 might not have a primary regulatory role and not be rate-limiting for iron transport in adult rats [76].

On the regional level the analyses are less specific for PN^+^/PN^−^-related properties because the WB and qRT-PCR protein and mRNA signals from glial cells, neuropil or interstitium superimpose with neuronal signals. Especially in PC and SUB the contribution from PN^+^ neurons may be in particular low since the percentage of PN^+^ neurons are only 4% and 5%, respectively (Figure 6). On the other hand, strong contributions to the signals are likely in SNpr and RN where the PN^+^-percentages are 45% and 86%, respectively.

Yet, the data reveal the investigated regions known to be rich in PN^+^ neurons have mostly a higher iron protein and mRNA level as the PN-poor entorhinal cortex. The PN and its associated bustling activity of iron proteins are probably the beneficial provisions that enable PN^+^ neurons to effectively ward off iron-induced oxidative stress. The correlation between the occurrence of a PN and reduced vulnerability has been demonstrated in multiple studies [13,15,16,17,22,23,24,25,26].

#### 3.1.2. PN^+^ Neurons May Have a Special Iron Household to Maintain a High Metabolic Rate

PNs often surround the soma and proximal dendrites of fast-spiking neurons for example in the cortex and medial nucleus of the trapezoid body [77]. In the cortex, 60–80% of the PN^+^ neurons belong to the group of parvalbumin-positive GABAergic interneurons, which are characterized by high-frequency action potential activity, i.e., fast-spiking neurons. Further, they are characterized by the expression of the high-voltage gated potassium channel subunit Kv3.1b [78].

As prerequisites, fast-spiking interneurons have unique electrophysiological properties and particularly high energy consumption. This is reflected by enrichment with mitochondria and cytochrome c oxidase, which most likely supports extensive membrane ion transport and GABA metabolism [79].

The average firing rate determines the metabolic cost with demand for ATP to maintain homeostasis of intracellular ion concentrations. Most energy was expended on reversing Na${}^{+}$ entry during action potentials and pumping Ca${}^{2+}$ out of the cell [80]. Neuronal activity, i.e., action potential generation, input integration, and synaptic transmission, accounts for 50–80% of this energy usage [81]. Potential energy is stored in transmembrane ion gradients, which creates a cellular battery whose maintenance and active restoring accounts for most of the brain’s ATP consumption. ATP producing enzymes require iron as a redox-active metal.

An increased iron-induced oxidative stress and ferroptosis are the backside of a high energy consumption. We conclude that highly active neurons need more iron, and thus need a special iron household and homeostasis, but also protective mechanism to defend excessive iron-induced oxidative stress and ferroptosis. The iron binding ability of the chondroitin sulfate proteoglycans of the PN and the cellular iron import and storage proteins ensure a sufficient capture of needed iron, but also PN^−^ and protein-mediated iron scavenging and discharging if needed.

#### 3.1.3. Limitations of the Study

The number of animals studied for the individual techniques may appear low. However, we directly compared the iron and iron protein content on the individual neuron level among PN^+^ and PN^−^ neurons. For SBC, i.e., iron protein content, the analysis was done on more than 8000 individual neurons for each investigated iron protein. For each of the two rats with more than 4000 analysed neurons per iron protein, the results were the same. For PIXE, i.e., iron concentration, 267 individual neurons were measured in two rats (153 and 114). The increase in the iron content of PN^+^ neurons vs. PN^−^ neurons were already significant for each classified subgroup of neurons for the individual rats. Intersubject variability did not affect the combined results.

Cytochrome c oxidase enzyme histochemistry on the cellular level was performed on the PC within the limits of a pilot study only. The results merely suggest that PN^+^ neurons have generally a higher cytochrome c oxidase activity, but corroborate the conclusion of an increased energy metabolism of PN^+^ neurons that were drawn from the analyses of the cellular iron and iron protein contents.

## 4. Materials and Methods

For the study 10 three months old male Wistar rats (*Rattus norvegicus f. domestica*) were used for quantitative mapping and analysis of the intracellular iron (n=2), IHC of iron proteins and PNs (n=1), quantitative analysis of the intracellular iron proteins (n=2), regional quantification of iron protein mRNA (n=2), antibody specificity approval and regional iron protein quantification (n=3). The animals were obtained from and housed at the then animal care facility of the Paul Flechsig Institute for Brain Research of Leipzig University. They were kept on a 12/12 h dark/light cycle with free access to food and water. Experiments were carried out in accordance to the guidelines of the European Council Directive (1986; 86/609/EEC) and with approval by the local authorities (T61/01, T63/09). All animals were anesthetized and sacrificed in a 5-liter anesthesia chamber by opening a 100% CO_2_ influx to a flow rate of 1 l/min.

### 4.1. Quantitative Element Mapping of Neurons in Brain Slices

#### 4.1.1. Preparation of Brain Sections

After rats were sacrificed, they were transcardially perfused with saline (0.9% NaCl)/ 0.1% heparin to eliminate hem-iron. Further, a fixative solution of 4% formaldehyde and 0.1% glutaraldehyde in 0.1 M PBS (pH 7.4) was transcardially perfused for 30 min. Brains were removed from the skull, cut into three coronal sections and post-fixated in the same fixative solution overnight at room temperature (RT). After dehydration in increasing ethanol concentrations and followed repletion in methylbenzoate, the samples were embedded in paraffin. Frontal sections of 5 μm thickness were cut containing PC (Bregma—4.1 mm ), SUB, and SN (Bregma—5.8 mm). The sections were transferred to Superfrost^®^ glass slides, deparaffinized with xylene, rehydrated in decreasing concentrations of ethanol and transferred into PBS (pH 7.4).

#### 4.1.2. Lectinhistochemistry and Embedding

To visualize PNs, brain slices were lectinhistochemically stained with biotinylated *Wisteria floribunda* agglutinin (Bio-WFA, Sigma-Aldrich, Burlington, MA, USA, 1:300) at 4 °C overnight. After washing in PBS-Tween (PBS-T; pH 7.4) and rinsing in Tris-HCl (pH 8) brain slices were incubated for 1 h at RT with peroxidase-conjugated streptavidin (ExtrAvidin^®^, Sigma-Aldrich, 1:1000) to reveal the lectin binding sites. The staining was enhanced by 3,3’-diaminobenzidine (DAB, Sigma-Aldrich) and Ni (nickel ammonium sulphate, purity grade 99.999%, Sigma-Aldrich) in Tris-HCl (pH 8). Brain slices were rinsed in Tris-HCl and PBS-T and dehydrated in alcohol. The brain sections, still on Superfrost^®^ object slides, were covered with a small droplet of mounting medium (DePeX, Serva, Heidelberg, Germany) that was spread out by shortly covering the sections with another object slide. Holding the object slide sandwich vertically, whereby the cover slide was allowed to move freely downward by gravity, a thin layer of embedding medium was produced after the cover slide eventually slipped off. After 24 h of drying at RT, a rectangular area of 20 mm × 15 mm containing the brain section was cut out, peeled off, and attached to aluminium frames using double-sided adhesive carbon tape. Light microscopic images (Olympus BX51, Hamburg, Germany) were taken for orientation and re-recognition of the cells within the PIXE element maps, which is especially necessary for the unstained neurons.

Nickel is used as an enhancer, because it is visible with light microscopy due to its black precipitate, and in element mapping due to its characteristic X-ray emission [82]. The Ni-staining was proven to not introduce any significant impurities, especially to not alter the distribution and concentration of iron [14,21,61,82].

#### 4.1.3. µPIXE Analysis

Quantitative element mapping and analyses were performed with a 1 μm proton beam of 2.25 MeV energy using the high energy ion nanoprobe LIPSION at Leipzig University, Felix Bloch Institute for Solid State Physics [14,83]. The proton beam was scanned over the brain sections while the induced X-rays emitted from the sample were recorded. This technique is called PIXE, the prefix “µ” in µPIXE refers to the capability of microscopic element mapping using a scanning focused beam. Quantitative analysis is based on (i) spectral deconvolution by least squares fitting of the element peaks and the underlying background, (ii) calculated yield to each element from fundamental parameters for x-ray production and matrix effects, and (iii) the theoretical description of the detector responses, geometric parameters, and absolute efficiencies [84]. The correct description of the detector system is verified by analysis of certified reference materials.

From the recorded X-rays, tagged with the position, overlap-free and quantitative element images were created using dynamic analysis [85], which is part of the GeoPIXE software. GeoPIXE also provides a wide range of graphical tools that were used to encircle the regions of interest (ROIs) in the images and determine the average elemental concentrations therein. Thus, 227 individual neurons were analysed. Since the MeV-protons cause relatively low background radiation in the element characteristic X-ray spectrum, the minimum detection limits, especially for elements of atomic number between *Z*=21…30, thus also for iron, are at microgram per gram level which corresponds to concentrations around 10 μM.

The significance of differences between elemental concentrations of PN^+^ and PN^−^ neurons was tested using the *t*-test with unequal sample size from two rats. The test of normal distribution was performed with a Q-Q-plot. µPIXE data between the rats did not differ significantly (*t*-test, *p* > 0.05).

### 4.2. Cellular Analysis of Iron Proteins

#### 4.2.1. Preparation of Brain Sections

Post-fixated brains were cryoprotected in 30% sucrose and 0.1% sodium azide in PBS. Frozen (−20 °C) frontal sections (30 μm thickness) of PC (Bregma—4.1 mm), SUB, RN and SN (Bregma—5.8 mm) were cut on a cryomicrotome (Reichert-Jung with Leica Frigocut). All sections were washed thoroughly in PBS for subsequent histochemical staining. All antibodies, markers and dilutions are listed in Table A1.

#### 4.2.2. Multiple Lectin-/Immunohistochemistry

We used multi-coloured fluorescence staining for the brain slices to (a) identify neurons, (b) detect the PNs, and (c) characterize the intracellular iron proteins in one section. For the comfortable visualization of the components with fluorescence microscopy, the colour labelling was chosen by visual prevalence of the microscopist’s eyes (in contrast to the next paragraph on quantification). Free-floating slices were treated with 2% BSA in PBS-T (pH 7.4) at RT for 1 h. The sections were then incubated at 4 °C overnight with a cocktail of three primary markers: (a) NeuN, (b) *Wisteria floribunda* agglutinin (WFA), (c) primary antibodies against Tf, TfR1, FtH, FtL, DMT1, MTP1, or DcytB (Table A1). One exception was for TfR were NeuN was substituted by DAPI because of an antibody interference. After repeated washing in PBS-T the brain slices were incubated in the dark for 1 h at RT with the secondary antibody cocktail including: (a) Cy5-conjugated antibody (excluded in the Tf mix), (b) Cy2-conjugated streptavidin, and (c) Cy3-conjugated antibody (Table A1). After repeated washing in PBS-T the samples stained for TfR were counterstained with DAPI (0.67 μM, Invitrogen, Carlsbad, CA, USA) for 20 min in the dark. All brain slices were finally rinsed in PBS-T and PBS, transferred to glass slides, dehydrated and mounted in DePeX^®^. The specificity of the antibodies was proven by negative IHC control staining and specific WB products (Figure 8A). For DcytB, an insufficient signal was obtained in WB and IHC and was therefore discontinued.

#### 4.2.3. Slide-Based Cytometry

SBC was used to quantify the iron metabolism related proteins in PN^+^ and PN^−^ neurons in multi-fluorescence-labelled brain slices (14 slices in total from two rats) [86]. The quantification occurs via the fluorescence signal intensity. The used iCys Research Imaging Cytometer (CompuCyte Corporation, Westwood, MA, USA) is equipped with three different lasers. We choose the argon ion laser (488 nm) for the recognition of the marked cells (Cy2-labelled) and the PNs (Cy3-labelled), and the helium-neon laser (633 nm) for the analysis of the iron proteins (Cy5-labelled). The separate excitation of the Cy5-labelled iron proteins excludes spillover of unspecific fluorescence light and ensures proper signal quantification. Therefore, the lectin-/immunohistochemistry protocol described above was adapted for the SBC. The cell marker NeuN was labelled by a Cy2-conjugated secondary antibody, and WFA was labelled with Cy3-conjugated streptavidin (Table A1). For the iron proteins FtH, FtL, DMT1, and MTP1 Cy5-conjugated secondary antibodies were used. For the TfR staining, an interfering antibody combination required the exclusion of NeuN-Cy2. It was replaced by NeuroTrace green Fluorescent Nissl Stain, which has the same excitation wavelength as Cy2. Additionally, because no reliable Cy5-conjugated anti-chicken-IgY antibody was available at this time, Tf was exceptionally labelled with Cy2 and NeuN with Cy5.

An orange filter (580/30) and a long red filter (650/LP) were used for the analysis of Cy3 and Cy5, respectively. For the Tf-Cy2 measurement a spillover from the Cy3 signal into the Cy2 channel was blocked by a green filter (530/30), and a potential rest spillover was excluded by a software-supported calculation comprising the control signal. Photomultiplier (PMT) generated digital images were obtained. Overview images with lower resolution (Figure 5A) were done to manually encircle the brain region of interest, to control autofocus, to set threshold triggers and to adjust the PMT amplification. All settings were saved and later re-applied for the corresponding brain region of the associated control slide. High-resolution automatic scans create a PMT generated mosaic image of the depict brain area. The image was further cytometrically analysed. Thereby, cells were automatically identified and displayed in a X-Y-coordinate dot plot. By setting specific criteria new dot plots were created displaying the classified subpopulations of PN^+^ and PN^−^ neurons. A final evaluation of the correct identification of the cells was done by browsing the gallery images for false classifications (Figure 5B). Both groups were analysed according to their iron protein related Cy5 fluorescence signal (Tf Cy2). The final statistical analysis and comparative of the intracellular iron proteins in PN^+^ and PN^−^ neurons is described in detail in Reinert et al., 2011 [86]. On average more than 8000 neurons were analysed per iron protein.

### 4.3. Cytochrome C Oxidase Enzyme Histochemistry

The increased activity of CytOx, the last enzyme of the respiratory chain, is a sign for a high metabolic rate. For its visualization enzyme histochemistry was performed (modified from [87]). Rats were sacrificed and transcardially perfused with a mixture of 2.5% formaldehyde, 1% glutaraldehyde and 4% sucrose in PBS (0.1 M, pH 7.4) for one hour. One hour after perfusion, during which time the rat was cooled on ice, the brain was removed from the skull and post-fixated in 4% formaldehyde and 20% sucrose in PBS at 4 °C overnight. Frozen (−20 °C) frontal sections were cut as described above and kept deep frozen until reaction. Therefore, sections were washed in PBS and reacted for CytOx by incubation in substrate solution containing cytochrome c (16.15 μM, Sigma-Aldrich), DAB (2.33 mM) and 4% sucrose in PBS. Incubation was done at RT under visual control, the reaction was stopped after 4 hours. Sections were subsequently double-stained for PNs (WFA-Cy3) as described above and observed under bright field and fluorescence mode.

### 4.4. Regional Analysis of Iron Proteins

To complement the cellular quantification of iron and iron proteins, we additionally quantified iron proteins and their encoding mRNAs in isolated brain tissue using WB and qRT-PCR. To reveal PN-related information also in the homogenate’s cell and matrix ensemble, PN-rich brain tissue (PC, SUB, RN, SN) was compared with PN-poor brain tissue (EC, Bregma—6.3). Therefore, anesthetized rats were decapitated and brains were removed and snap-frozen in liquid nitrogen. Selected brain regions were dissected on dry ice and powderized in a 2 mL tube.

#### 4.4.1. Quantitative Real-Time PCR

The weighted samples were homogenized on ice in an according amount of RLT-buffer and carrier RNA (QIAGEN, Düsseldorf, Germany). They were then transferred to MinElute spin columns (QIAGEN, Hilden, Germany). Total RNA was isolated using the RNeasy Mikro Kit (QIAGEN) according to the manufacturer’s protocol. Isolated RNA (6 μL of each sample) was transcribed in cDNA using the SuperScriptTM III First-Strand Synthesis Super-Mix (Invitrogen). The cDNA was amplified by qRT-PCR (Rotor-Gene 2000, Corbett Research, Mortlake, Australia) using the primer listed in Table 2, supplemented with HotStarTaq Mix (QIAGEN) and SYBR-Green I (Invitrogen). The RNA sample load was normalized with housekeeping gene *β*-actin. Standard curves of serial dilutions from total RNA were used to transform the Ct (cycle of threshold) values into concentration values by applying the comparative ΔΔCT method [88,89,90]. The amplification products were verified by electrophoresis (BioRad, Feldkirchen, Germany) using a 2% agarose gel in Tris-acetate-EDTA buffer (pH 8.3). Samples were loaded with 50% glycerol/1 mM EDTA (Sigma-Aldrich), 0.25% bromphenol blue (Fluka, Buchs, Switzerland), 0.25% xylencyanol (Sigma-Aldrich) and SmartLadder (Eurogentec, Cologne, Germany). Gel was bathed in ethidium bromide (0.5 mg/L, Sigma-Aldrich) for 15 min in the dark and products were detected by 312 nm UV light (Biometra, Göttingen, Germany).

#### 4.4.2. Protein Extraction and Western Blotting

The grounded and weighted brain tissue samples were homogenized on ice in 9 volumes (*w*/*v*) protein extraction buffer B (20 mM Tris-HCl (pH 7.2), 150 mM NaCl, 2 mM MgCl_2_, 2 mM EDTA, 2 mM EGTA, 1% NP40, 5 mM NaF, 1 mM Na_3_VO_4_, 5% glycerol, 1 mM PMSF, 1 μg/mL leupeptin, cOmplete protease inhibitor cocktail (Roche, Basel, Switzerland)) using an Ultra-Turrax^®^ disperser (IKA, Staufen, Germany). The homogenates were centrifuged at 50,000× *g* for 30 min at 4 °C and supernatants were filled into new tubes. Protein concentrations were determined after the Bradford method and equalled for all samples with *d*H_2_O to 20 μg protein/µL. For each region of interest, homogenates were pooled, loaded as triplicate on a SDS polyacrylamide gradient gel (4–15%) and proteins were separated under reducing conditions. Proteins were further transferred to PVDF membranes (Polyscreen, DuPont, Wilmington, DE, USA) at 4 °C over night in a tank blot system (TV400, biostep, Burkhardtsdorf, Germany). Blots were blocked for 1 h in blocking buffer (TBS-T, 2% BSA, 0.05% Tween 20) and incubated with primary antibodies (Table A1) in 2% BSA at 4 °C overnight. Blots were washed in TBS-T and probed with horseradish peroxidase (HRP) conjugated secondary antibodies (Table A1), diluted in 1% BSA, for 1 h at RT. Chemoluminescence was visualized on a Kodak Image Station 2000R using ECL substrate (Lumigen TMA-6, GE Healthcare, Chicago, IL, USA). The blots were stripped (0.2 M glycine, pH 2.1, 1% Tween 20, 0.1% SDS) for 2 h at RT, and blotted again for *β*-actin. The semi-quantitative analysis of the iron proteins and the housekeeping gene thus occurred on the same membrane and was done densitometrically using the TINA 2.09 software.

## 5. Conclusions

PN-ensheathed neurons constitute a neuronal subpopulation characterized by a distinctly higher iron content and higher levels of the iron proteins transferrin, transferrin receptor, and metal transport protein 1 (ferroportin). This characteristic reflects an iron metabolism adopted for maintaining a fast energy conversion to power a high neuronal activity. Furthermore, the physicochemical properties of the perineuronal net support the high firing activity by ion spacial buffering [21], which also reduces the iron-induced oxidative stress in the neuronal microenvironment. We hypothesize that PN-ensheathed neurons represent a special group of resilient pacemaker neurons characterized by a bustling iron metabolism with outstanding iron handling capabilities.

To understand the low vulnerability of PN^+^ neurons to degenerative processes, it is further required to elucidate how the iron metabolism of PN^+^ and PN^−^ neurons react to pathological or age-related brain iron accumulations.

Eventually, one might speculate that in general, a higher neuron activity with the increased energy metabolism entails the adopted iron protein household, which in turn improves the handling of excess iron. This would explain the-well known protective strategy: Keep your brain active.

## Figures and Tables

**Table 1:** Summary of the results that demonstrate higher iron protein levels in PN^+^ neurons (left) and summary of the regional iron protein (middle) and mRNA analyses (right). Note: The regional analyses also include non-neuronal sources of protein/mRNA, which reduce the PN^+^ effect, especially for regions with low PN^+^ contents (PC, SUB). ↑ increase, ↓ decrease, ∼ no difference.

	Cellular: PN^+^ vs. PN^−^ Neurons						Regional: PN-Rich Region vs. PN-Poor Region (EC)								
	Slide-Based Cytometry						Western Blot						qRT-PCR		
	Tf	Tf-R	FtH	FtL	MTP1	DMT1	Tf	Tf-R	FtH	FtL	MTP1	DMT1	Tf	Tf-R	FtH
**PC**	↑	↑	↑	↑	↑	↑	∼	↑	↑	↑	∼	∼	∼	∼	∼
**SUB**	↑	↑	∼	↑	↑	↑	∼	↑	↑	↑	∼	∼	↑	∼	∼
**RN**	↑	↑	∼	∼	↑	∼	↑	↑	↑	↑	↑	∼	↑	↑	↑
**SN**	↑	↑	∼	∼	↑	∼	↑	↑	↑	↑	∼	↓	↑	↑	↑

**Table 2:** Primer (Metabion, Planegg/Steinkirchen, Germany) used for qRT-PCR. β-actin was amplified as reference. Primer were designed with NCBI and Primer3.

	5${}^{\prime }$-Primer-3${}_{\mathrm{forward}}^{\prime }$	5${}^{\prime }$-Primer-3${}_{\mathrm{reverse}}^{\prime }$	Size
Tf	AGATGGAGGTGGAGATGTGG	GAGAGCCGAACAGTTGGAAG	312 bp
TfR	CCTGAGGGTTATGTGGCATT	ATGGGGGAAACTGAGTATGG	286 bp
FtH	CCTGGAGTTGTATGCCTCCT	GTGCACACTCCATTGCATTC	236 bp
DMT1	CTCCACCATGACTGGAACCT	CAGCCTATTCCGTTGGAGAA	273 bp
MTP1	GGGTGGATAAGAATGCCAGA	TGCTCCTGTTTTCTCCTGCT	261 bp
DcytB	GTCATGGGCATGATCTTCCT	GGTGGCACCAAAAGTGT	228 bp
β-Actin	AGCCATGTACGTAGCCATCC	CTCTCAGCTGTGGTGGTGAA	280 bp

## Data Availability

The datasets analysed during this study are available from the corresponding author on request.

## Abbreviations

The following abbreviations are used in this manuscript:

Fe	iron
Ni	nickel
PN(s)	perineuronal net(s)
PN^+^	PN-ensheathed
PN^−^	PN-less
SBC	slide-based cytometry
PIXE	particle-induced X-ray emission
Tf	transferrin
TfR	transferrin receptor
DMT1	divalent metal transporter 1
MTP1	metal transport protein 1
DcytB	duodenal cytochrome B
WFA	*Wisteria floribunda* agglutinin
PC	parietal cortex
SN	substantia nigra
SUB	subiculum
RN	red nucleus
EC	entorhinal cortex
CytOx	cytochrome c oxidase
SEM	standard error

## Appendix A

**Table A1:** Antibodies/markers used for the (lectin-/immuno)histochemical detection, and SBC and WB quantification. ^1^ biomol, ^2^ Santa Cruz, ^3^ adi, ^4^ Sigma-Aldrich, ^5^ Chemicon, ^6^ invitrogen, ^7^ Jackson ImmunoResearch Laboratories, ^8^ abcam, ^9^ Dako, ^10^ Pierce, ^★^ 100 μM stock solution.

Target Antigen/Marker	Host^conj.^	Dilution 1:x		RRID/Cat#
		WB	IH	
**PRIMARY Reaction**				
**iron proteins**				
human transferrin (plasma protein)	chicken	500	250	^1^ A80-156A
rat transferrin receptor, CD71	mouse	500	150	^2^‘RRID:AB-1120369
human ferritin H (Y-16)	goat	500	200	^2^ RRID:AB-2107172
human ferritin L (Y-20)	goat	500	200	^2^ RRID:AB-2107158
rat DMT1+IRE	rabbit	500	150	^3^ RRID:AB-1620356
mouse MTP1	rabbit	500	150	^3^ RRID:AB-1619475
mouse DcytB	rabbit	500	150	^3^ RRID:AB-1614641
**PN marker**				
GalNAc	*WFA* ^biotin^		300	^4^ A1516
**cell marker**				
β-actin	mouse	20,000		^4^ RRID:AB-476697
NeuN	mouse		150	^5^ RRID:AB-2298772
NeuroTrace fluor. Nissl stain ^green^			1000	^6^
DAPI			15,000 ^★^	^6^
**SECONDARY Reaction**				
goat	donkey^Cy5^		200	^7^
rabbit	donkey^Cy5^		200	^7^
rabbit	donkey^Cy3^		300	^7^
rabbit	donkey^Cy2^		250	^7^
chicken	goat^Cy3^		350	^8^
chicken	donkey^Cy2^		400	^7^
mouse	donkey^Cy5^		200	^7^
mouse	donkey IgM^Cy5^		200	^7^
mouse	donkey^Cy3^		300	^7^
mouse	donkey^Cy2^		250	^7^
mouse	donkey IgM^Cy2^		300	^7^
biotin	Streptavidin^Cy3^		250	^7^
rabbit	donkey^biotin-SP^		1000	^7^
biotin	ExtrAvidin^HRP^		1000	^4^
chicken	rabbit^HRP^	10,000		^4^
goat	rabbit^HRP^	10,000		^9^
mouse	goat^HRP^	10,000		^10^
rabbit	goat^HRP^	10,000		^10^
